# Internet videoconferencing delivered cognitive behavior therapy for generalized anxiety disorder: protocol for a randomized controlled trial

**DOI:** 10.1186/s13063-022-06520-5

**Published:** 2022-07-23

**Authors:** Vesna Trenoska Basile, Toby Newton-John, Bethany M. Wootton

**Affiliations:** Discipline of Clinical Psychology, Graduate School of Health, University of Technology Sydney, PO Box 123 Broadway, Ultimo, NSW 2007 Australia

**Keywords:** Generalized anxiety disorder, GAD, Cognitive behavioral therapy, Remote treatment, Randomized controlled trial, RCT, Videoconference, Protocol

## Abstract

**Background:**

Generalized anxiety disorder (GAD) is a chronic mental health condition that results in a significant individual and societal burden. While cognitive behavioral therapy (CBT) is well established as an efficacious treatment for GAD, many patients experience logistical barriers when accessing face-to-face CBT. Remotely delivered treatments remove many of these barriers. Despite emerging evidence demonstrating the efficacy of remotely delivered CBT for GAD, studies examining the efficacy of remote methods for GAD that are analogous to standard face-to-face treatment, in particular synchronous treatments such as CBT delivered via online videoconferencing (VCBT), are needed.

**Methods:**

The authors propose a two-group randomized controlled trial comparing the efficacy of VCBT for GAD against a waitlist control group. The recruitment target will be 78 adults with primary GAD of at least moderate severity. The manualized high-intensity VCBT intervention will be delivered weekly over a 10-week period. After treatment completion, waitlist participants will receive the same VCBT delivered in a brief format (i.e., weekly over a 5-week period). Treatment for both groups will be delivered in real time via an online teleconferencing platform. Outcome measures will be administered at baseline, mid-treatment, post-treatment, and 3-month follow-up.

**Discussion:**

This trial will report findings on the efficacy of a remote synchronous high-intensity VCBT intervention for GAD. The results have the potential to contribute towards advancing our knowledge on the evidence base for GAD, as well as increase the dissemination of VCBT for GAD.

**Trial registration:**

Australian New Zealand Clinical Trials Registry ACTRN12621000786897. Registered on 22 June 2021

## Introduction

Generalized anxiety disorder (GAD) is characterized by excessive and uncontrollable worry that is accompanied by a number of physical and/or cognitive symptoms [1]. The disorder is often chronic [22] and results in considerable individual and economic burden [27, 48]. GAD can be effectively treated with cognitive behavioral therapy (CBT) [11]; however, numerous barriers to accessing treatment exist including cost, difficulty accessing a trained clinician, and geographical isolation [10, 16, 37, 40]. One way to overcome these barriers is to provide specialized treatment remotely, using digital health technologies.

Remotely delivered treatments do not require the clinician and the client to be in the same location and these interventions can be provided in either a low-intensity or high-intensity fashion [54]. Low-intensity remote treatments involve the client working through largely self-help materials either online or via a workbook, accompanied by brief asynchronous clinician contact (i.e., 10 min per week by telephone or email). High-intensity remote treatments involve using digital health technologies to provide synchronous sessions that are analogous to standard face-to-face treatment. While low-intensity remote treatments have been demonstrated to be efficacious in the treatment of GAD, with several studies demonstrating medium to large pooled effects across studies [2, 43], there is limited evidence examining high-intensity remote treatments for this condition [50]. The literature that does exist has considerable limitations (i.e., uncontrolled studies with small samples sizes) [42]. Thus, the efficacy and acceptability of high-intensity remote CBT for GAD requires further investigation.

One promising remote high-intensity approach to treatment includes Internet videoconferencing-delivered CBT (VCBT). VCBT involves the therapist and client working together over video-link, maintaining the visibility of the therapist, and clients’ non-verbal behaviors. Recent research has demonstrated that high-intensity remote CBT results in equivalent outcomes compared to traditional face-to-face treatment across a number of common mental health disorders [51]. While there has been little research investigating the efficacy of VCBT for GAD specifically, case study results have been promising. For example, Bouchard and Renaud [5] demonstrated that VCBT resulted in significant improvements from pre-treatment to post-treatment in GAD symptoms. Similar results were found in a case study by Théberge-Lapointe et al. [47], and this study further demonstrated that the effects of VCBT were durable, with results maintained up to 12 months post-treatment [47]. Importantly, the therapeutic alliance does not appear to be affected when CBT is delivered in this format [5, 52].

This study describes the procedures and methodology of a randomized controlled trial (RCT) investigating the efficacy and acceptability of VCBT for GAD. Based on the limited existing literature, it is hypothesized that high-intensity VCBT will (1) be acceptable to individuals with GAD; (2) result in significant reductions in symptoms, resulting in large within-group at pre-treatment and follow-up and large between-group effect sizes at 3-month follow-up; and (3) brief VCBT will result in outcomes similar to those seen in standard length face-to-face treatment. It is anticipated that the results of the proposed study will inform best-practice psychological treatment for GAD.

## Materials and method

### Participants

Seventy-eight individuals will be recruited for this study. To be included in the trial, participants are required to (1) be an Australian resident, (2) be aged 18 or above, (3) meet criteria for GAD as the primary disorder, (4) experience symptoms of at least “moderate severity,” and (5) be on a stable dose of psychotropic medication. Participants will be excluded if they have symptoms that will put them at risk of harming themselves or others or will confound results of the treatment. Participants will also be excluded if they do not have regular access to the Internet and camera. A complete list of inclusion and exclusion criteria is outlined in Table 1.

**Table 1:** Study inclusion and exclusion criteria with rationale

Inclusion criteria	Rationale
1. Australian resident	Study population
2. Aged 18 or above	Study population
3. Fluent in English	Treatment confound/participant concern
4. Meet criteria for GAD as primary disorder and disorder is of at least “moderate severity” (defined as a score of 4 on the DIAMOND module severity measure)	Study population
5. Medication free or on a stable dose of psychotropic medication	Treatment confound
6. Not currently receiving regular psychological services for their GAD symptoms (defined as sessions at least once a week with a qualified mental health professional)	Treatment confound
Exclusion criteria	Rationale
1. Severe depressive symptoms as assessed by a score of 20 or above on the PHQ-9	Participant safety
2. Are at suicide risk as assessed by a score of “2” (more than half the days) or higher on item 9 of the PHQ-9 on the screening questions or via clinician judgment during the telephone interview using the C-SSRS	Participant safety
3. Engage in daily alcohol use or daily illicit drug use	Treatment confound
4. Presence of a schizophrenia spectrum disorder as assessed by the DIAMOND	Treatment confound
5. Significant cognitive/intellectual impairment as assessed during diagnostic interview	Treatment confound
6. Medical condition that may interfere with treatment	Treatment confound
7. Do not have access to a computer with a camera and stable internet on a regular basis	Feasibility

### Design

A CONSORT-R compliant 2-group RCT superiority trial will be used to investigate the research questions. Participants will be randomly assigned to an immediate treatment group (*n* = 39) or a waitlist control group (*n* = 39). Group 1 will receive immediate access to a manualized high-intensity VCBT intervention. Group 2 will receive treatment after group 1 completes treatment. Given this is the first controlled trial investigating VCBT for GAD, a waitlist control group was considered appropriate. The content and design of this RCT are in accordance with the guidelines for clinical trial protocols as specified by the Standard Protocol Items: Recommendations for Interventional Trials (SPIRIT) 2013 statement [9]. The SPIRIT checklist was followed to ensure compliance.

### Recruitment

Participants will be recruited via advertising on social media, posts on professional networking sites, and direct email/letter to community-based clinicians, general practitioners, and psychiatrists. Hardcopy flyers will be posted on community noticeboards. Interested participants will complete a two-stage screening process to assess eligibility criteria involving, firstly, an online screening process, followed by a telephone interview. Interested participants will initially be directed to an online screening questionnaire consisting of a participant information sheet and consent form, demographic questionnaire, and symptom screeners. Participants are informed in the participant information sheet and consent form that de-identified data may be used for ancillary studies. Participants who meet criteria based on the online screening questionnaire will then complete a diagnostic interview via telephone to confirm their diagnostic status and assess comorbid conditions. Telephone interviews will be audio-recorded to determine interrater reliability. Eligible participants will then be randomized to one of the two groups. Allocation sequence will be sequentially numbered based on completion date of screening interview and randomization will be conducted by the chief investigator (BW) using a random number generator.

### Screening self-report measures

#### Demographic questionnaire

A 15-item standard demographic questionnaire will be used to collect self-reported information on age, location, gender, marital, employment and education status, medication use, and access to technology required for the study.

#### Risk Questionnaire

Risky behaviors including deliberate self-harm and regular alcohol and/or illicit drug use will be assessed with the Risk Questionnaire, a 5-item questionnaire that has been used as a screening tool in other remote CBT treatment studies [55].

#### DIAMOND screener [49]

The DIAMOND screener is a 30-item self-report questionnaire that indicates to the clinician which disorders from the DSM-5 require further investigation. Participants who endorse GAD items on the DIAMOND screener will progress onto the second screening stage.

### Screening interview

#### Diagnostic Interview for Anxiety, Mood, and Obsessive-Compulsive and Related Neuropsychiatric Disorders [49]

The DIAMOND is a structured clinical interview that systematically assesses the DSM-5 diagnostic criteria for anxiety disorders, mood disorders, obsessive-compulsive and related disorders, trauma- and stressor-related disorders, schizophrenia spectrum disorders, eating disorders, somatic symptom and related disorders, substance use disorders, and selected neurodevelopmental disorders. The DIAMOND demonstrates very good interrater reliability (kappa = .71) and test-retest validity (kappa = .68) for the GAD diagnosis [49].

#### Columbia-Suicide Severity Rating Scale (C-SSRS) [41]

The C-SSRS is a standardized assessment of suicide risk and can be used to measure the severity of suicidal ideation and behaviors [41]. The scale assesses (1) the severity of suicidal ideation, (2) the intensity of suicidal ideation, (3) suicidal behaviors, and (4) lethality [41]. The scale demonstrates sound psychometric properties [34, 41] and has been used in multiple settings including emergency departments [7], juvenile justice [26] and veterans affairs [32].

### Primary outcome measure

#### Generalized Anxiety Disorder Questionnaire-7 item (GAD-7) [46]

The GAD-7 is a 7-item measure of symptoms of generalized anxiety disorder. Each of the seven items are rated on a 4-point scale from 0 *(not at all)* to 3 *(nearly every day)* and a total score is calculated by summing each of the seven items*.* The scale has demonstrated good psychometric properties in previous samples [21, 24, 46]. A score of 10 or above indicates clinically significant symptoms of generalized anxiety disorder [46]. The GAD-7 will be used as the primary outcome measure.

### Secondary outcome measures

#### Generalized Anxiety Disorder Dimensional Scale (GAD-D) [31]

The GAD-D is a 10-item measure of generalized anxiety symptoms. Participants rate the frequency with which they have experienced GAD symptoms over the past month on a 5-point Likert scale ranging from 0 (never) to 4 (all of the time), resulting in a total score ranging between 0 and 40. Previous studies have established acceptable psychometric properties [31].

#### Penn State Worry Questionnaire-3 item (PSWQ-3) [4]

The PSWQ-3 is a 3-item, self-report questionnaire designed to assess the core features of worry in GAD (uncontrollability, excessiveness, and multiple worry domains). Participants rate items on a 5-point scale and responses are summed, with higher scores indicating greater worry. The PSWQ-3 has demonstrated good psychometric properties in previous samples [4].

#### Overall Anxiety Severity and Impairment Scale [39]

The OASIS is a 5-item transdiagnostic self-report measure of anxiety symptoms. The OASIS has been shown to have strong psychometric properties in previous studies [6, 39], and a cut score of 8 [8] has been used to indicate clinically significant anxiety symptoms in previous studies.

#### Patient Health Questionnaire-9 item (PHQ-9) [28]

The PHQ-9 is a 9-item measure of depressive symptoms. Each item is assessed on a 4-point Likert scale from 0 (*not at all*) to 3 (*nearly every day*) and symptoms are assessed over the previous 2 weeks. Scores are summed and total scores ≥ 10 are used to indicate clinically significant depressive symptoms [35] with 88% sensitivity and 88% specificity [28]. The PHQ-9 has been demonstrated to have excellent psychometric properties in previous samples [28, 58].

#### Intolerance of Uncertainty Scale (IUS-12) (Carleton et al., 2007)

The IUS-12 is a 12-item self-report questionnaire measuring responses to uncertainty, ambiguous situations, and the future. The 12 items are rated on a 5-point Likert scale ranging from 1 (not at all characteristic of me) to 5 (entirely characteristic of me). The IUS-12 has demonstrated robust psychometric properties in community (Fergus & Wu, 2013) and treatment-seeking samples (Shihata et al., 2018).

#### Core Beliefs Questionnaire (CBQ)-Trait version [53]

The CBQ is a 17-item measure of core beliefs. It instructs participants to rate how much they believe each belief item (e.g., “I am unlikeable”) on a 6-point Likert scale from 1 (strongly disbelieve) to 6 (strongly believe). Higher scores indicate greater endorsement of negative core beliefs about the self. The CBQ-Trait version has demonstrated adequate validity and reliability and shown to have excellent internal consistency (Cronbach’s *α* = .96) [53].

#### Clinical Perfectionism Questionnaire (CPQ) [15]

The CPQ is a widely used 12-item measure of perfectionism. Participants are asked to rate the degree to which each item describes them over the past month on a scale from 1 (*not at all*) to 4 (*all of the time*). The CPQ has been shown to have acceptable reliability and validity in both clinical and community samples [12, 14].

#### NIMH Clinician Global Impression (CGI) Scale (self-report version) [17]

The CGI is a commonly used single-item measure of severity of symptoms and improvement in symptoms. Severity scores range from 1 (normal) to 7 (severely ill) and improvement scores range from 1 (very much improved) to 7 (very much worse). The CGI has been shown to be a valid and reliable clinical outcome measure in previous studies [3, 56].

#### Sheehan Disability Scale (SDS) [45]

The SDS is a commonly used 5-item measure that assesses how much psychiatric symptoms have interfered with work, social, and home life functioning. A cut score of 5 on any subscale has been used to identify individuals with clinically relevant symptoms in previous studies [33].

### Process/acceptability measures

#### Working Alliance Inventory-Short Form Revised (WAI-SR) [20]

The WAI-SR is a shortened version of the Working Alliance Inventory (WAI [23];. It is used to measure the therapeutic alliance in an ongoing client-therapist interaction. It comprises 12 items that are scored on a 5-point Likert scale, ranging from “seldom” to “always.” The WAI-SR has been shown to have high internal consistency, with a Cronbach’s *α* of 0.91 [20, 38] and high reliability, with test-retest reliability of 0.93 (95% CI 0.83 to 0.97) [18].

#### Client Satisfaction Questionnaire (CSQ) [30]

The CSQ is an 8-item measure of the participant’s satisfaction with the treatment they were provided. The scale has demonstrated adequate psychometric properties in previous studies [25, 30]. A score of 22 or above has previously been used to indicate adequate satisfaction with treatment [25].

#### Acceptability Questionnaire (AQ)

The AQ is a 10-item measure of acceptability of remote treatments. The questionnaire has been used in other remote treatments [55].

The time points for administration of each of the measures are outlined in Table 2. Participants will complete the self-report measures online using REDCap [19]. The link to these questionnaires will be emailed to participants. Participants will complete the diagnostic interview via telephone or Internet videoconferencing. While the full DIAMOND will be administered at baseline, only the GAD module will be administered at post-treatment and 3-month follow-up. The DIAMOND will be administered by trained interviewers who are either provisionally registered or fully registered psychologists under the supervision of an experienced clinical psychologist.

**Table 2:** Administration schedule for outcome measures

	Screening	Pre-treatment	Mid-treatment	Post-treatment	3-month follow-up
Screening measures					
*Demographics*	+				
*Risk questionnaire*	+				
*DIAMOND screener*	+				
*DIAMOND Interview*	+			+	+
*C-SSRS*	+				
Primary outcome measure					
*GAD-7*		+	+	+	+
Secondary outcome measures					
*GAD-D*		+	+	+	+
*PSWQ-3*		+	+	+	+
*OASIS*		+	+	+	+
*PHQ-9*		+	+	+	+
*IUS-12*		+	+	+	+
*CBQ*		+	+	+	+
*CPQ*		+	+	+	+
*CGI*		+	+	+	+
*SDS*		+	+	+	+
Process/acceptability measures					
*WAI-SF*			+	+	
*CSQ*			+	+	
*AQ*			+	+	

*Note*. *DIAMOND* Diagnostic Interview for Anxiety, Mood, and Obsessive-Compulsive and Related Neuropsychiatric Disorders, *C-SSRS* C-Suicide Severity Rating Scale, *GAD-7* Generalized Anxiety Scale-7 item, *GAD-D* Generalized Anxiety Disorder Dimensional Scale, *PSWQ-3* Penn State Worry Questionnaire-3 item, *OASIS* Overall Anxiety Severity and Impairment Scale, *PHQ-9* Patient Health Questionnaire-9 item, *IUS-12* Intolerance of Uncertainty Scale-12 item, *CBQ* Core Beliefs Questionnaire, *CPQ* Clinical Perfectionism Questionnaire, *CGI* NIMH Clinician Global Impression Scale (self-report version), *SDS* Sheehan Disability Scale, *WAI-SF* Working Alliance Inventory-Short Form, *CSQ* Client Satisfaction Questionnaire, *AQ* Acceptability Questionnaire

### Treatment

Treatment will be provided at a university outpatient clinic in Australia and will follow a manualized VCBT intervention which is informed by the Intolerance of Uncertainty Model of GAD [13, 44]. Such CBT interventions have been found to be efficacious in previous clinical trials for GAD [29]. Those in the immediate treatment condition will receive 10 weekly (50 min) treatment sessions to be conducted via Zoom [57]. The treatment for this group will comprise six modules and will cover the following: (1) psychoeducation, (2) cognitive restructuring to challenge positive beliefs about worry, (3) behavioral experiments to develop a greater tolerance to uncertainty, (4) problem solving training to reduce negative problem orientation, (5) imaginal exposure to address cognitive avoidance, and (6) relapse prevention. The treatment protocol is outlined in Table 3. Participants will also be required to complete homework tasks between sessions. When the immediate treatment group concludes treatment, the control group will receive a brief manualized version of the same treatment taking place over 5 weeks (i.e., 5 weekly, 50-min sessions). The same interventions will be covered; however, a smaller number of sessions will be dedicated to each intervention (see Table 3). After the post-treatment assessment, participants are encouraged to consult with the primary care physician if they require ongoing treatment for their symptoms of GAD or other mental health conditions.

**Table 3:** Treatment protocol

Standard treatment		Brief treatment	
Session	Module	Session	Module
1	Psychoeducation	1	Psychoeducation
2	Cognitive restructuring	2	Cognitive restructuring and problem solving training
3	Cognitive restructuring	3	Behavioral experiments
4	Problem solving training	4	Imaginal exposure
5	Cognitive restructuring and problem solving training	5	Relapse prevention
6	Behavioral experiments		--
7	Behavioral experiments		--
8	Imaginal exposure		--
9	Imaginal exposure		--
10	Relapse prevention		--

Treatment will be delivered by provisionally registered or fully registered psychologist(s) under the supervision of an experienced clinical psychologist. Treating psychologists will be in their final year of a Master of Clinical Psychology degree at the University of Technology Sydney. All treating psychologists will be familiar with delivering manualized treatments and thoroughly trained in the administration of the treatment protocol by the project investigators. All sessions will be recorded and at least 10% of sessions will be randomly selected for treatment compliance and integrity checking. Treating clinicians will receive weekly supervision to review client progress and address clinical issues arising from sessions.

### Data storage and analysis

In order to maintain confidentiality, all electronic data (including session recordings and other identifiable information) will be stored on a password-protected computer that is only accessible to members of the research team. All hardcopy data will be stored in a locked filing cabinet in the chief investigators locked office.

Group differences in demographic data and pre-treatment measures will be analyzed with independent samples *t*-tests with Bonferroni-corrected *p-*values (continuous measures) and *chi*-square tests (categorical measures). Treatment acceptability will be examined using descriptive statistics. The main analyses comparing the treatment group to the control group will be carried out using conservative intention-to-treat principles and using mixed-linear models with an unstructured covariance structure. Multiple imputation will be used to handle missing data. Effect sizes using Cohen’s *d* will be calculated for within-group and between-group differences, based on pooled standard deviations for both the entire sample using the estimated marginal means and completer sample (i.e., those who completed post-treatment and 3-month follow-up questionnaires). All analyses will be conducted based on the total score of the relevant outcome measure. The efficacy and acceptability of the brief treatment will be examined in the same manner described above. Comparisons between standard treatment and brief treatment will be analyzed using benchmarking analyses using the procedure outlined by Minami et al. [36]. All analyses will be performed using IBM SPSS Statistics (version 26). Results will be disseminated via national and international conference presentations, as well as in peer-reviewed journal articles. Participants are able to access publications resulting from the study by contacting the chief investigator.

### Power

With alpha set at 0.05, power set at 0.80, and a sample size of 34 in each group, the study is powered to enable the detection of large effect size (i.e., Cohen’s *d* = 0.80) differences in symptoms, which would be the minimum expected reduction in the RCT based on existing research [11]. Therefore, 39 individuals will be recruited in the immediate treatment group and 39 individuals in the waitlist control group, in order to hedge against attrition. Therefore, the total sample size for the study is 78.

### Ethical approval and trial registration

The study was approved by the University of Technology Sydney Health and Medical Research Ethics Committee (UTS HREC REF NO. ETH21-5843). The trial is registered with the Australian and New Zealand Clinical Trials Registry (ACTRN12621000786897) and includes the full study protocol and participant information sheet and consent form. While not anticipated, any changes to the protocol will be updated through the ANZCTR registry.

## Discussion

GAD is a chronic and impairing mental health condition [22]. CBT is effective for GAD [11]; however, many individuals experience logistical barriers to accessing this treatment [10, 16]. High-intensity VCBT overcomes many of these barriers and may assist in the dissemination of evidence-based treatment for GAD. The primary aim of this study is to examine the acceptability and efficacy of VCBT for GAD. A secondary aim is to examine the acceptability and efficacy of a brief VCBT treatment for GAD. This will be the first study to examine the efficacy of VCBT for GAD using a controlled design. Therefore, the results of this study may inform how to best deliver VCBT for GAD. It is anticipated that the results will contribute to the growing evidence base that remotely delivered high-intensity CBT (whether standard or brief in length) is a viable option for individuals who are unable to access face-to-face treatment.

### Trial status

The protocol version number is v1.0 which was approved on 22 July 2021. Recruitment will commence in October 2021 and is expected to be completed by July 2023.

## Data Availability

De-identified data will be made available to other researchers upon reasonable request.
